# Effects of Renal Denervation on Cardiac Structural and Functional Abnormalities in Patients with Resistant Hypertension or Diastolic Dysfunction

**DOI:** 10.1038/s41598-017-18671-6

**Published:** 2018-01-19

**Authors:** Shiying Wang, Suxia Yang, Xinxin Zhao, Jun Shi

**Affiliations:** Department of Nephrology of Huaihe Hospital of Henan University, Henan Province, China

## Abstract

The aim of the present study is to systematically evaluate the impact of RDN on cardiac structure and function in patients with resistant hypertension (RH) or diastolic dysfunction. We retrieved Pubmed, Embase and Cocharane Library databases, from inception to April 1^st^, 2016. Studies reporting left ventricular mass (LVMI) or left ventricular (LV) diastolic function (reflected by the ratio of mitral inflow velocity to annular relaxation velocity [E/e’]) responses to RDN were included. Two randomized controlled trials (RCTs), 3 controlled studies and 11 uncontrolled studies were finally identified. In observational studies, there was a reduction in LVMI, E/e’ and N-terminal pro B-type natriuretic peptide (BNP) at 6 months, compared with pre-RDN values. LV ejection fraction (LVEF) elevated at 6 months following RDN. In RCTs, however, no significant change in LVMI, E/e’, BNP, left atrial volume index or LVEF was observed at 12 months, compared with pharmaceutical therapy. In summary, both LV hypertrophy and cardiac function improved at 6 months after RDN. Nonetheless, current evidence failed to show that RDN was superior to intensive (optimal) drug therapy in improving cardiac remodeling and function.

## Introduction

Hypertension is the most common risk factor for cardiovascular diseases^1^, which are the leading causes of death worldwide^2^. Resistant hypertension (RH) is defined as blood pressure (BP) above target despite the optimal use of at least 3 different classes of antihypertensive drugs (including a diuretic)^3^. Patients with RH are at increased risk for major cardiovascular events^4,5^. Left ventricular hypertrophy (LVH) is a common cardiac structural change in hypertension, and considered a more important risk factor than BP level itself^6^. The structural alternations accounts for LV diastolic abnormalities, which can be detected early in hypertensive heart diseases^7,8^. Like LVH, LV diastolic dysfunction has been associated with increased cardiovascular mortality^9^. Therefore, improvement of LVH or diastolic dysfunction is an important treatment target in RH patients.

Sympathetic nerve system (SNS) plays a crucial role in the development of RH and LVH, and is considered a potential target. Catheter-based renal denervation (RDN), a novel interventional technique to reduce renal afferent and efferent sympathetic nerve activity, has been proven effective in reducing BP in certain patients^10,11^. The effect of RDN on cardiac structure and function has also been studied in several small clinical studies^7,12^, while the results remains controversial. The aim of the present study is to systematically evaluate the impact of RDN on cardiac structure and function in patients with RH or diastolic dysfunction, by collecting currently available clinical evidences.

## Methods

### Data Sources and Searches

This systematic review and meta-analysis was conducted in accordance with the Preferred Reporting Items for Systematic Reviews and Meta-Analyses (PRISMA) statement^13^. We electronically retrieved Pubmed, Embase and Cocharane Library, from inception to April 1^st^, 2016, using the keywords as follows: “left ventricular dimensions”, “atrial dimensions”, “cardiac hypertrophy”, “cardiac dimensions”, “echocardiography”, “magnetic resonance imaging”, “cardiac imaging”, “echocardiogram”, “ventricular dysfunction”, “renal denervation” and “renal sympathetic denervation”. Related references of retrieved articles were also searched for potential eligibility. No language restriction was applied in the search process. However, we only included English-written full text in the final review. The whole search process was performed by two investigators independently.

### Study Selection

Observational studies reporting left ventricular mass (LVMI, indexed to body surface area) or LV diastolic function (reflected by the ratio of mitral inflow velocity to annular relaxation velocity [E/e’]) before and after RDN were included. Also, randomized controlled trials (RCTs) that comparing the effect of RDN with that of pharmaceutical therapy (PT) on LVM or diastolic function were involved. Detailed inclusion criteria were: 1) studies using cardiac imaging, namely echocardiography or cardiac resonance imaging, to assess LVM or diastolic function; 2) studies with no less than 10 subjects; 3) follow up of at least 6 months. Conferences abstracts, reviews and case reports were excluded. We checked the authors, methods and results to identify duplicate reports, which were excluded unless they featured different follow-up durations.

### Data extraction and quality assessment

After eligible articles being identified, data were extracted by two separate researcher. The characteristics of included studies, involving study design, sample size, measurements, study population and follow-up interval were extracted. Also, baseline characteristics of included participants, including age, gender, commodity diseases, body mass index, and usage of antihypertensive drugs were collected. The outcomes of interest, i.e. change in LVMI or diastolic function (including LVMI or diastolic function before and after RDN) were extracted. Any disagreement was resolved by consensus. The primary endpoints were: 1) LVMI and E/e’ change following RDN in observational studies; the difference in LVMI and E/e’ change between RDN and DT in RCTs. Secondary endpoints were: 1) LV ejection fraction (LVEF) response; 2) left ventricular diastolic diameter (LVDD) response; 3) left atrial volume (LAVI) response; 4) BNP response. Data at different time points were collected separately.

For observational studies, the mythological quality was assessed by means of the Newcastle-Ottawa scale. (Supplementary Table 1). For RCTs, Cochrane Collaboration Risk of Bias Tool was applied in the quality assessment. (Supplementary Table 2).

### Data Synthesis and Analysis

For observational studies, including controlled and single-arm studies, the changes in LVMI and E/e’ before versus after RDN were pooled. For RCTs, the differences in LVMI and E/e’ changes with RDN and PT were pooled and analyzed. The same strategy was used to handle secondary outcome parameters. Weighted mean difference (WMD) with 95% confidence interval (CI) was taken as treatment effect measure when outcome measurements in all trials were made on the same scale. The standard mean difference (SMD) was applied when the trials all assessed the same outcome, but measured it in a variety of ways (referring to Cochrane handbook). I^2^ was calculated and used to assess the between-study heterogeneity. I^2^ value of 25%, 50% and 75% represents low, moderate and high heterogeneity, respectively. A Fixed-effects model was applied unless the level of heterogeneity reached high. Data were mainly presented as mean ± standard deviance (SD), thus when only 95% CI or standard error (SE) was reported, we converted 95% CI or SE to SD according to the Cochrane handbook. P < 0.05 was considered statistically significant. We performed data analyses by the mean of Review Manager (version 5.2). Meta-regression analyses were conducted in STATA software (version 11.0) to evaluate the relationship between changes in LV remodeling or function and blood pressure.

## Results

**Figure 1:**
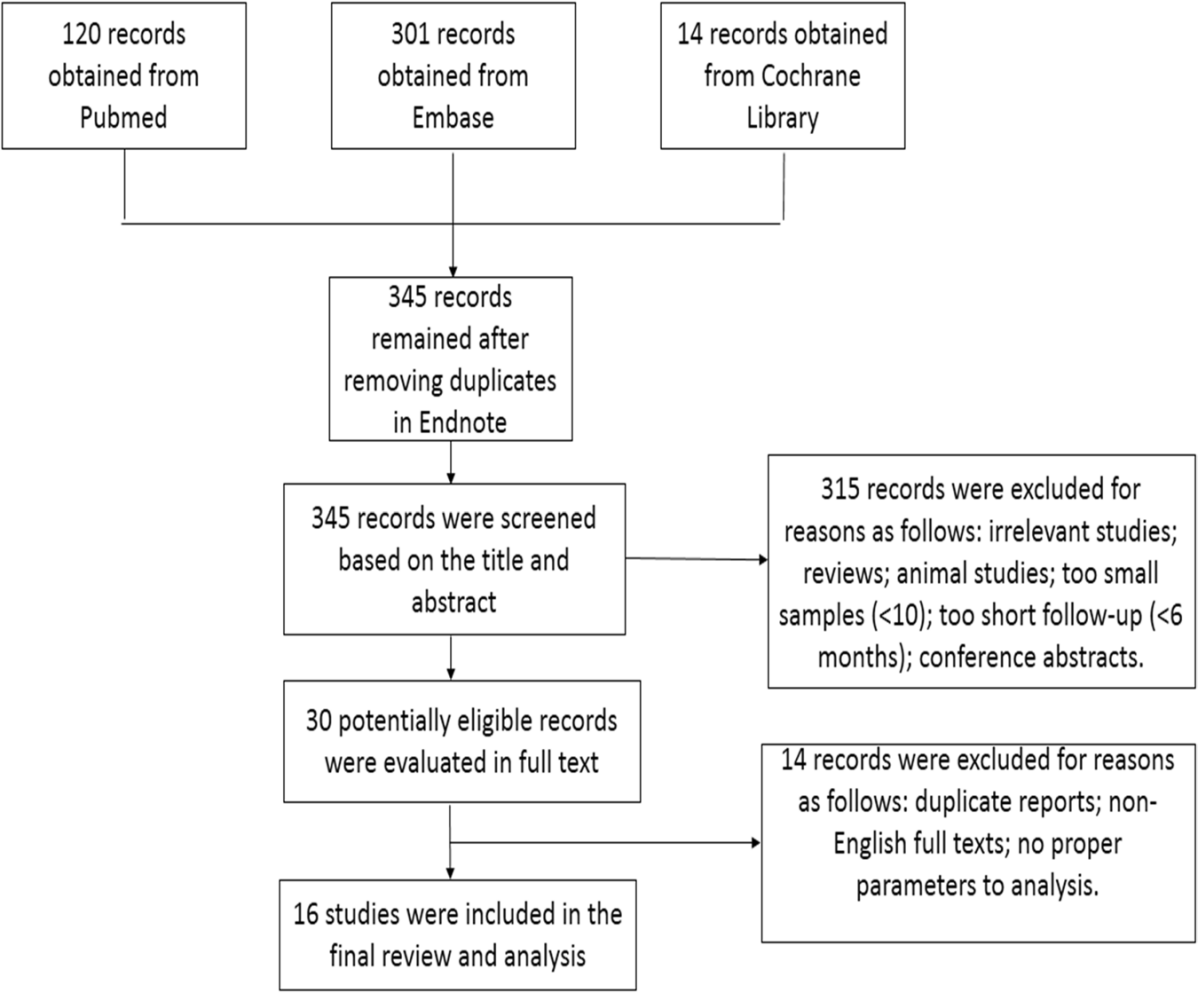
Flow chart of literature search.

Our primary search identified 435 records, while only 16 of these articles were finally included in our analysis (Fig. 1). There are 14 observational studies^7,12,14–25^ (including 11 uncontrolled studies and 3 controlled studies) and 2 RCTs^26,27^. The baseline characteristics of included subjects was shown in Table 1. Table 2 summarized details regarding studies with 6-months follow-up (number of medications, responses to medications, blood pressure control and inclusion criteria or the time when RDN started). Mean age varied from 53.9 to 74.6 years. Number of participants ranged from 14 to 100. Mean number of antihypertensive drugs varied from 4.3 to 6.4. Echocardiography was the most used technology (15 out of 16), six studies employed cardiac magnetic resonance (CMR), and 3 trials used both echocardiography and CMR. Most observational studies featured a follow up of 6 months, while both RCTs had a follow up of 12 months. Differing from other studies, the study by Patel et.al included HF patients with preserved EF (HFpEF)^26^. As shown in Supplementary Tables 1 and 2, most studies had a low risk of bias.

**Table 1:** Baseline characteristics of included studies and subjects.

First Author	Year	Treatment	N	Age(yrs)	Male	Diabetes	CAD	BMI	FU(months)	Imaging
Schirmer	2015	RDN	66	63.5 ± 1.2	36(55)	23(35)	14(21)	29.4 ± 0.6	6	Echo
Ewen	2015	RDN	30	61.9 ± 9.9	18(60)	8(32)	6(24)	30.4 ± 4.4	6	Echo
Verloop	2015	RDN	54	58 ± 10	27(50)	8(15)	9(17)	29.2 ± 5.2	12	CMR
Berukstis	2016	RDN	16	54.9 ± 7.9	9(56)	5(31)	5(31)	34.16 ± 4.02	6	Echo
Dorr	2015	RDN	100	65.4 ± 10.1	57(57)	38(38)	NR	NR	6	Echo
Ripp	2015	RDN	60	NR	NR	NR	NR	NR	6	Echo
Dores	2014	RDN	22	62.7 ± 7.6	17(50)	22(65)	7(21)	30.9 ± 5.3	6	Echo
McLellan	2015	RDN	14	64 ± 9	10(67)	2(14)	2(14)	31 ± 3	6	Echo/CMR
de Sousa	2016	RDN	31	65 ± 7	15(48.4)	22(71)	10(32)	31.8 ± 5.5	12	Echo
Tsioufis*	2016	RDN	17	57 ± 9	11(65)	6(35)	NR	33.79 ± 5.49	12	Echo
Kiuchi	2016	RDN	45	53.9 ± 11.3	26(58)	15(33)	6(13)	30.2 ± 4.3	6	Echo
Mahfound	2014	RDN	55	65 ± 10	39(71)	26(47)	NR	29.2 ± 4.3	6	CMR
		PT	17	70 ± 9	10(59)	7(41)	NR	28.6 ± 5.3	6	CMR
Tsioufis	2015	RDN	18	56 ± 10	12(67)	6(33)	NR	33.6 ± 5.4	6	Echo
		PT	10	54 ± 8	6(60)	3(30)	NR	31.8 ± 2.8	6	Echo
Brandt	2012	RDN	46	63.1 ± 10.2	31(67)	21(46)	20(44)	28.6 ± 3.4	6	Echo
		PT	18	63.0 ± 15.3	11(61)	7(39)	7(39)	28.1 ± 3.8	6	Echo
Patel	2016	RDN	17	74.1 ± 6.8	11(64.7)	8(47)	5(29)	30.5 ± 4.6	12	Echo/CMR
		PT	8	74.6 ± 4.8	4(50)	2(25)	1(13)	30.8 ± 7.4	12	Echo/CMR
Rosa	2016	RDN	52	56 ± 12	40(77)	12(22)	3(6)	31.2 ± 4.3	12	Echo
		PT	54	59 ± 9	34(63)	9(17)	4(7)	33.4 ± 4.7	12	Echo

Values are mean ± SD or n (%).

RCT: randomized controlled trial; RDN: renal denervation; PT: pharmaceutical therapy; N: number of patients; CAD: Coronary artery disease; BMI: body mass index (kg/m^2^). Echo: echocardiography; CMR: cardiac magnetic resonance. NR: not reported.

**Table 2:** Medications, treatment subjects and blood pressure control of included studies with 6-month follow-up.

First Author	No. of antihypertensive Drugs	No. of Patients Used Diuretics	Treatment Subjects (The time when RDN started)	Change in SBP (mmHg)
Schirmer	4.3 ± 0.1	66(100)	Patients scheduled for RDN for treatment of resistant hypertension (defined as office systolic blood pressure [SBP] >140 mm Hg)	−21.6*
Ewen	5.0 ± 1.6	23(92)	Patients with resistant hypertension (office SBP of at least 140 mmHg despite treatment with three or more antihypertensive drugs of different classes, including a diuretic at the maximum or highest tolerated dose)	−10*
Berukstis	6.44 ± 0.96	16(100)	Patients with suspected resistant hypertension	−16.2
Dorr	5.2 ± 1.2	99(99)	Patients with at least three antihypertensive medications of different classes, including diuretics, at the maximum tolerated doses and with office SBP >160 mm Hg (>150 mm Hg, type 2 diabetes mellitus) or ABPM > 135 mm Hg.	−11.4*
Ripp	NR	NR	Patients with blood pressure over 160/100 mmHg, and administration of at least three antihypertensive drugs in full doses plus a diuretic.	−11.1
Dores	5.8 ± 1.0	NR	With resistant hypertension	−5
McLellan	4.9 ± 1.8	14(100)	patients with treatment-resistant hypertension (defined as BP greater than goal target despite concurrent use of at least 3 antihypertensive medications)	−11
Tsioufis	4.5 ± 0.6	17(100)	Patients with resistant hypertension	−19*
Kiuchi	4.7 ± 1.2	45(100)	Resistant hypertensive CKD patients	−50.8*
Mahfound	4.6 ± 1.6	46(84)	Patients with an office systolic blood pressure (SBP) above goal (≥140 mmHg) or mean ambulatory 24-h SBP 0.135 mmHg despite the use of ≥3 antihypertensive agents of different classes, including a diuretic at maximum or highest tolerated doses	−22*
Brandt	4.7 ± 0.5	46(100)	Patients had an office BP of 160 mm Hg (150 mm Hg for type 2 diabetes patients) or more, despite treatment with at least 3 antihypertensive drugs (including a diuretic), with no changes in medication for a minimum of 3 months before enrollment.	−27.8*

Values are mean ± SD or n (%).

No.: number; RDN: renal denervation; SBP: systolic blood pressure; NR: not reported. *Indicates that the change is significant (p < 0.05).

### Impact of RDN on cardiac structure

**Figure 2:**
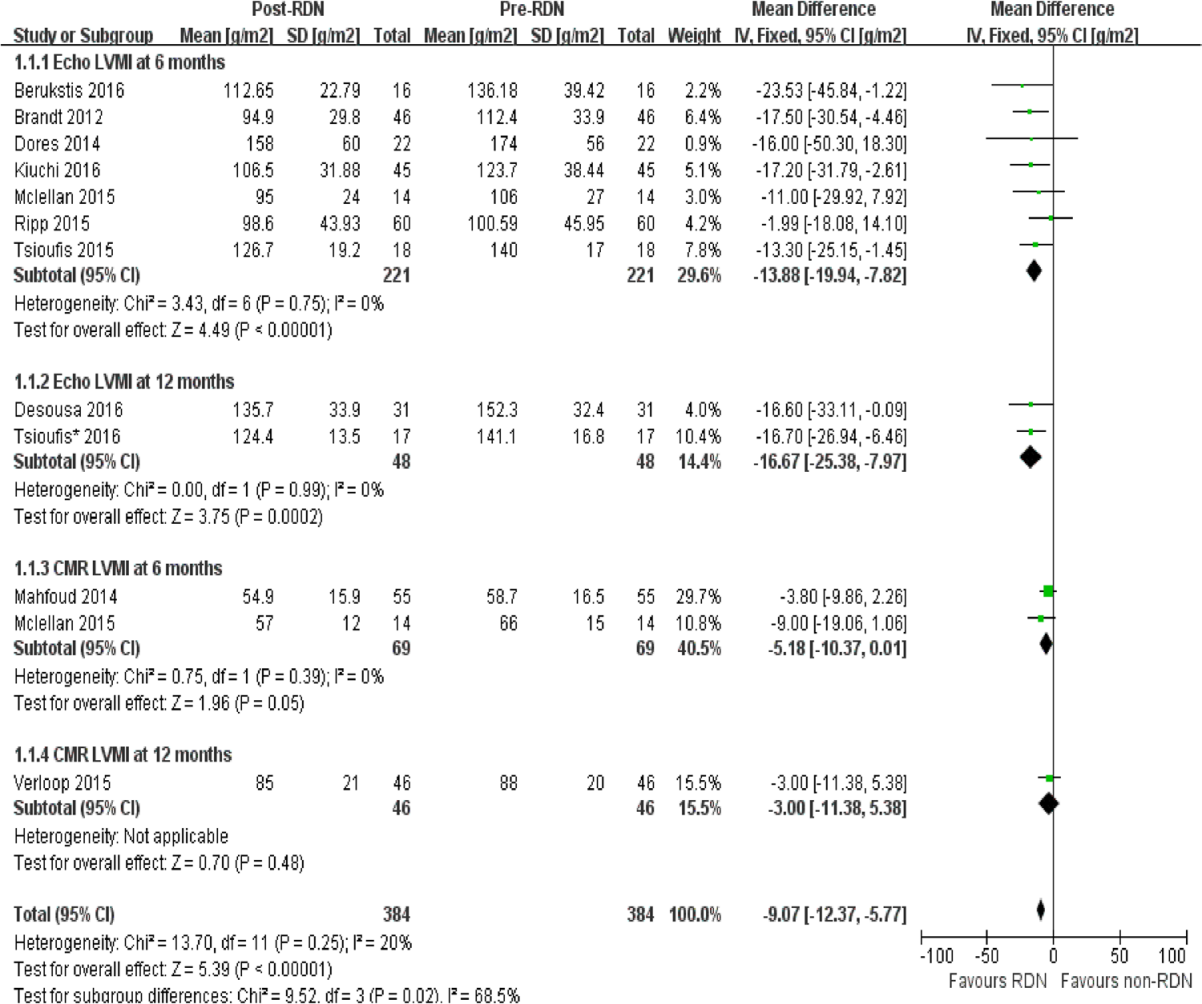
Forest plot of RDN changing LVMI in observational studies, stratified by follow up duration and imaging.

In observational studies, echocardiography showed that LVMI was reduced at 6 months following RDN (WMD = −13.88 g/m^2^, 95% CI = −19.94 to −7.82 g/m^2^, I^2^ = 0). More pronounced reduction was observed at 12 months (WMD = 16.67 g/m^2^, 95% CI = −25.38 to −7.97 g/m^2^, I^2^ = 0) (Fig. 2). CMR showed a reduction in LVMI at 6months (WMD = 5.18 g/m^2^, P = 0.05) but not 12 months (P = 0.48).

**Figure 3:**
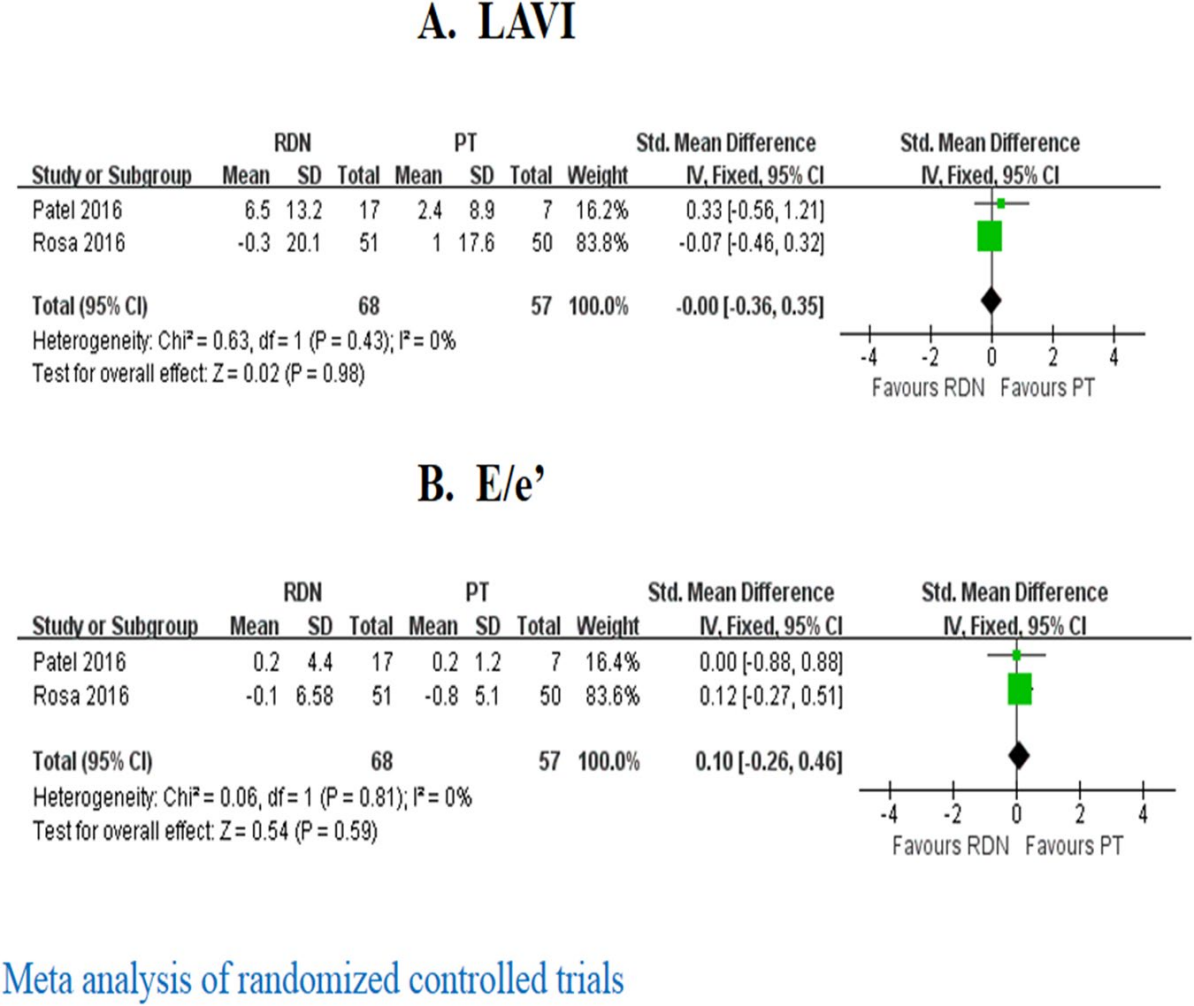
Forest plot of RDN changing LAVI (**A**) and E/e’ (**B**) at 12 months in randomized controlled trials.

In both RCTs, the difference in LVMI change between RDN and PT was not significant (Table 3). With regards to LAVI change, pooled analysis did not show significant between-group difference either (SMD = 0.00, 95% = −0.36 to 0.35, I^2^ = 0%) (Fig. 3A).

**Table 3:** Changes in cardiac structure and function from baseline to 12 months in randomized controlled trials.

		LVMI	E/e’	BNP (ng/L)	EF	LVDD (mm)	LAVI (ml/m^2^)
Rosa *et al*.	Change from baseline in RDN	−1.6 ± 14.4 (g/m^2.7^)	−0.1 ± 6.6	NR	0.01 ± 0.07	−0.6 ± 4.3	−0.3 ± 20.1
	Change from baseline in PT	−4.0 ± 11.1(g/m^2.7^)	−0.8 ± 5.1	NR	0.02 ± 0.11	−1.4 ± 5.6	1.0 ± 17.6
	Difference in change (P value)	P = 0.36	P = 0.58	NR	P = 0.61	P = 0.42	P = 0.71
Patel *et al*.	Change from baseline in RDN	0.7 ± 4.3 (g/m^2^)	0.2 ± 4.4	−3 (−59, 33)	NR	NR	6.5 ± 13.2
	Change from baseline in PT	0.2 ± 3.2 (g/m^2^)	0.2 ± 1.2	18 (−2, 30)	NR	NR	2.4 ± 8.9
	Difference in change (P value)	P = 0.807	P = 0.962	P = 0.559	NR	NR	P = 0.504

Data are presented as mean ± standard deviation or median (quartile 1, quartile 3). LVMI: left ventricular mass; E/e’: ratio of mitral inflow velocity to annular relaxation velocity; LLVDD: left ventricular diameter in diastolic; BNP: B-type natriuretic peptide; EF: ejection fraction; LAVI: left atrial volume index. Other abbreviations as in Table 1.

**Figure 4:**
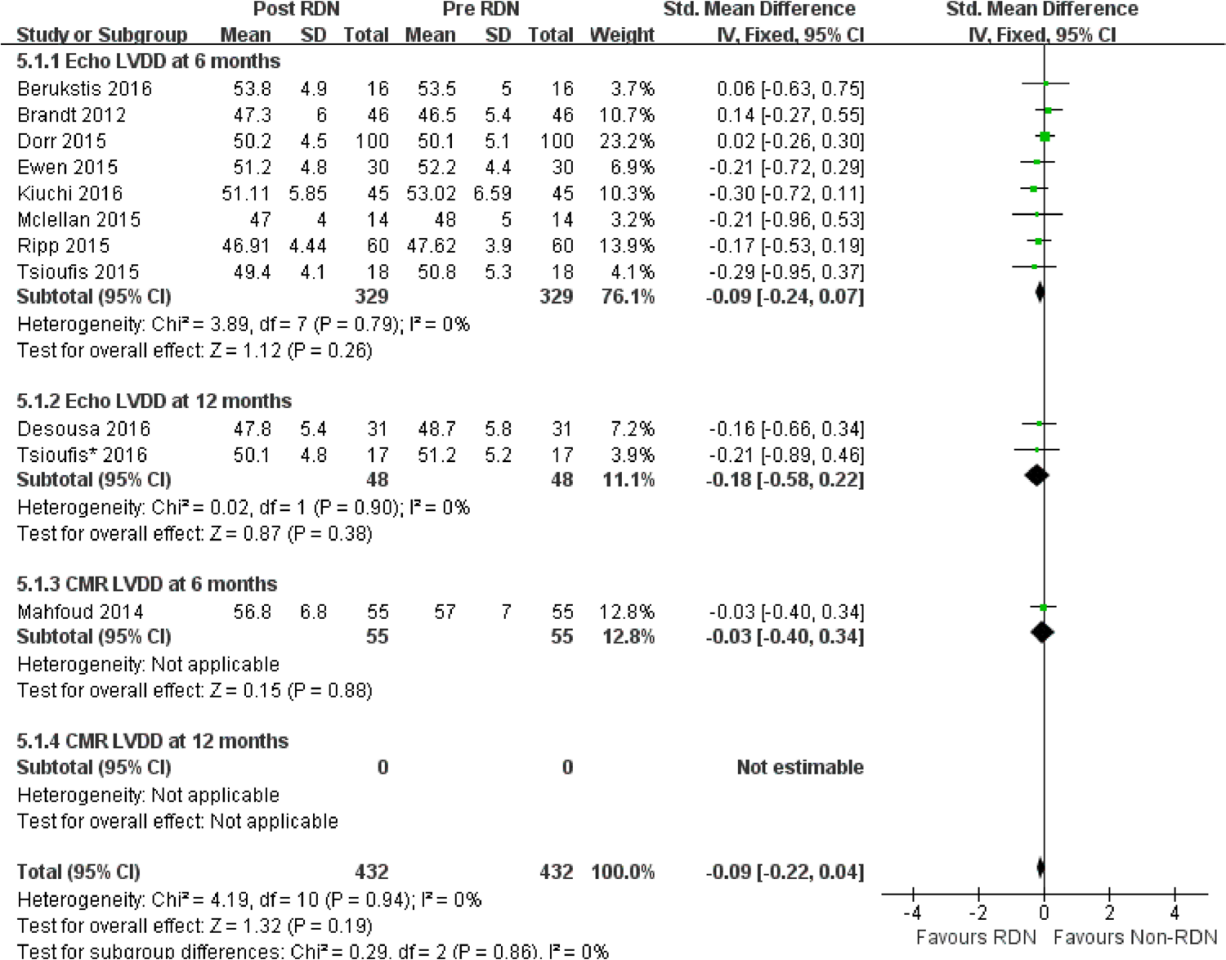
Forest plot of RDN changing LVDD in observational studies, stratified by follow up duration and imaging.

LVDD was not significantly changed after RDN in observational studies (Fig. 4). The difference in LVDD change between RDN and PT was not significant in the RCT by Patel *et al*. (Table 3).

### Impact of RDN on cardiac function

**Figure 5:**
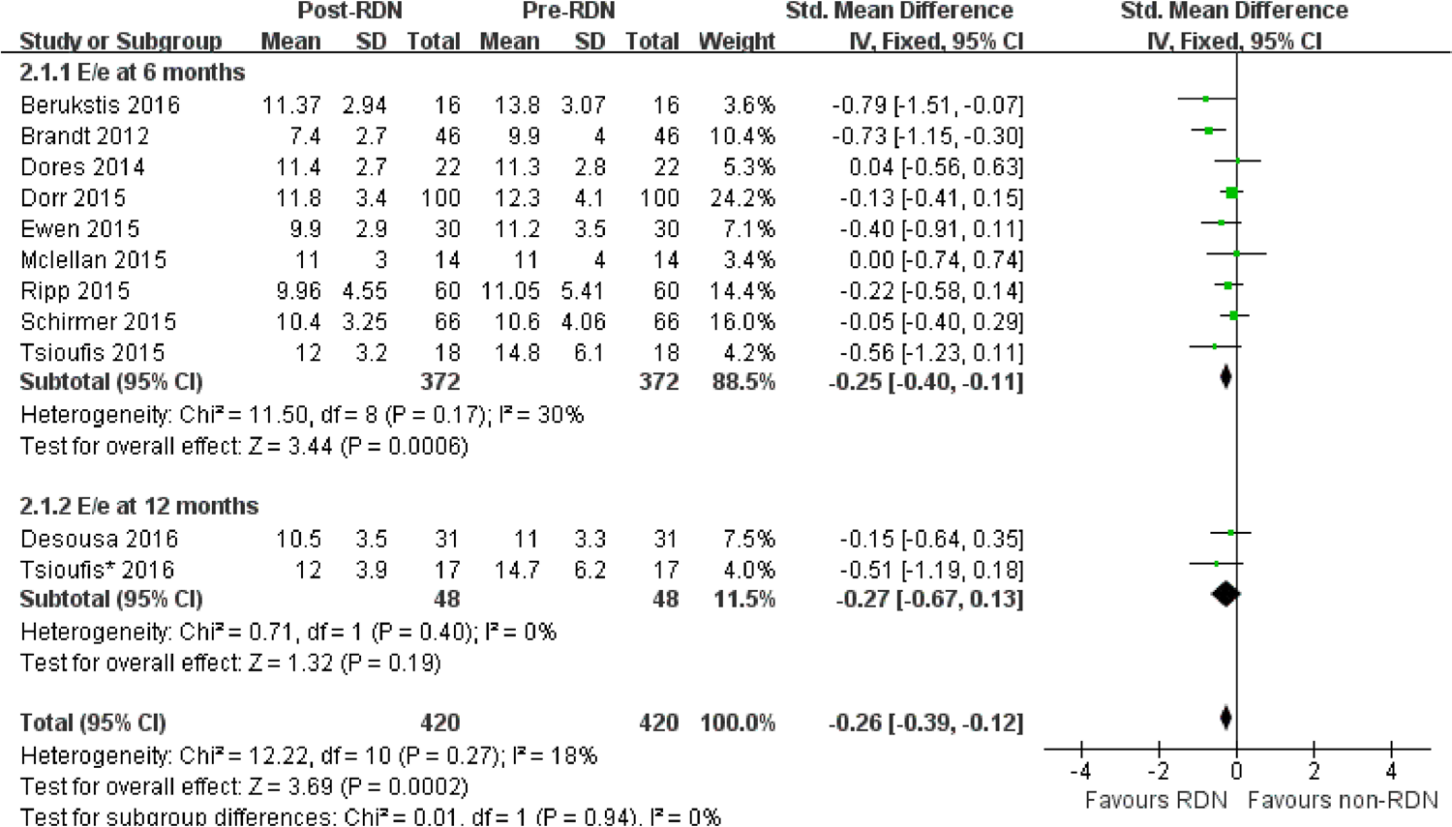
Forest plot of RDN changing E/e’ in observational studies, stratified by follow up duration.

In observational studies, pooled analysis revealed that E/e’ was significantly reduced at 6 months (SMD = −0.25, 95% = −0.40 to −0.11 I^2^ = 30%) but not 12 months (SMD = −0.27, 95% CI = −0.67 to 0.13, I^2^ = 0%) after RDN (Fig. 5). In RCTs, no significant difference was observed in E/e’ change between RDN and PT (SMD = 0.1, 95% CI = −0.26 to 0.46, I^2^ = 0) (Fig. 3B).

**Figure 6:**
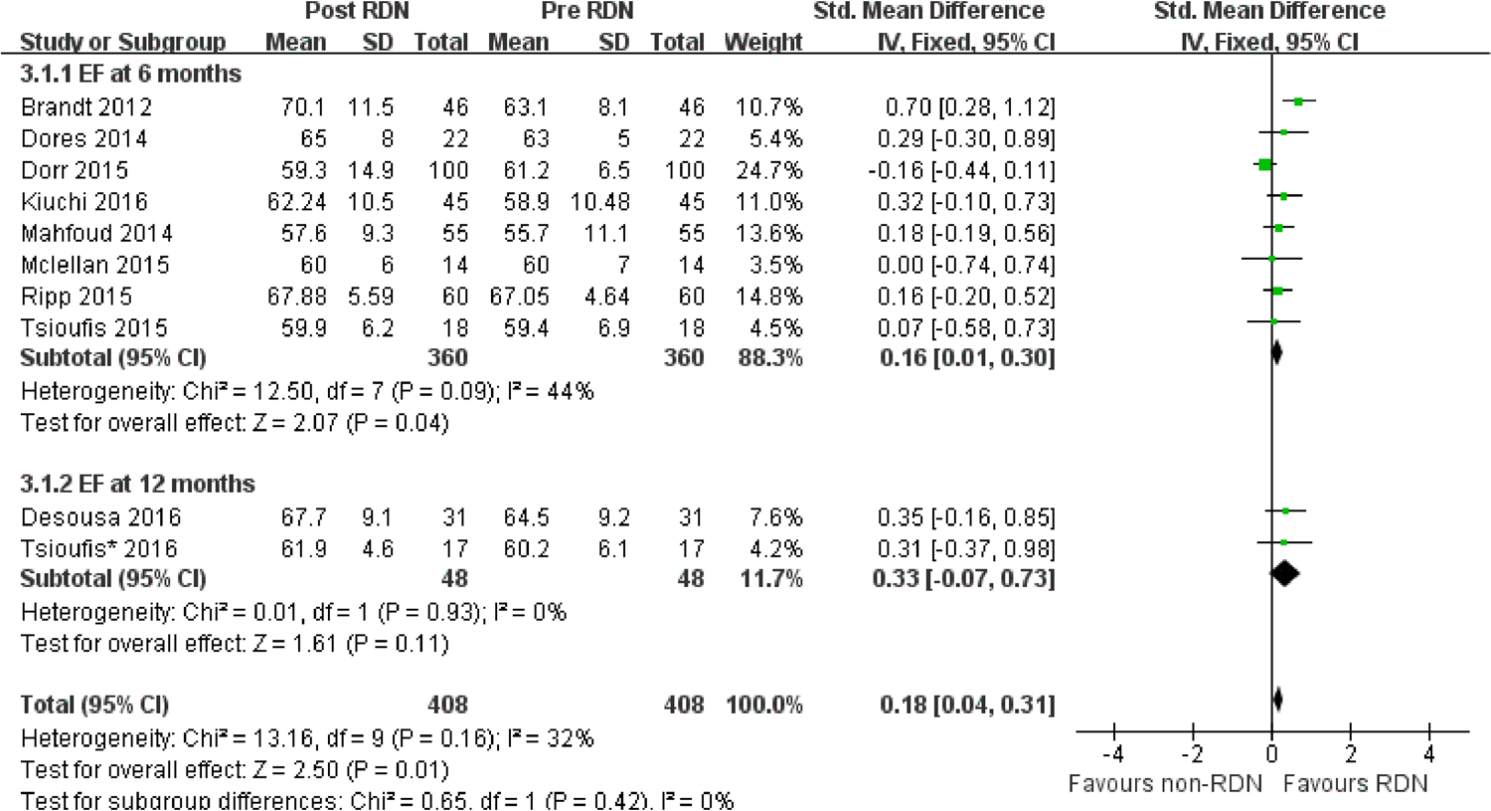
Forest plot of RDN changing EF in observational studies, stratified by follow up duration.

In observational studies reporting EF, pooled data showed that EF increased at 6 months (SMD = 0.16. 95% CI = 0.01 to 0.3, I^2^ = 44%) but not 12 months (SMD = 0.33. 95% CI = −0.07 to 0.73, I^2^ = 0) following RDN (Fig. 6). The RCT by Rosa *et al*.^27^ involving EF change showed no difference between RDN and PT (Table 2).

Two observational studies reported data regarding BNP^7,22^, pooled analysis showed a reduction in BNP (SMD = −0.35, 95% CI = −0.7 to 0, I^2^ = 39%) at 6 months following RDN (Supplementary Fig. 1). The RCT of Patel *et al*., which included HFpEF patients^26^, compared the BNP change in RDN group with that in PT group, and no significant difference was observed (Table 3).

### Meta-regression analysis

**Figure 7:**
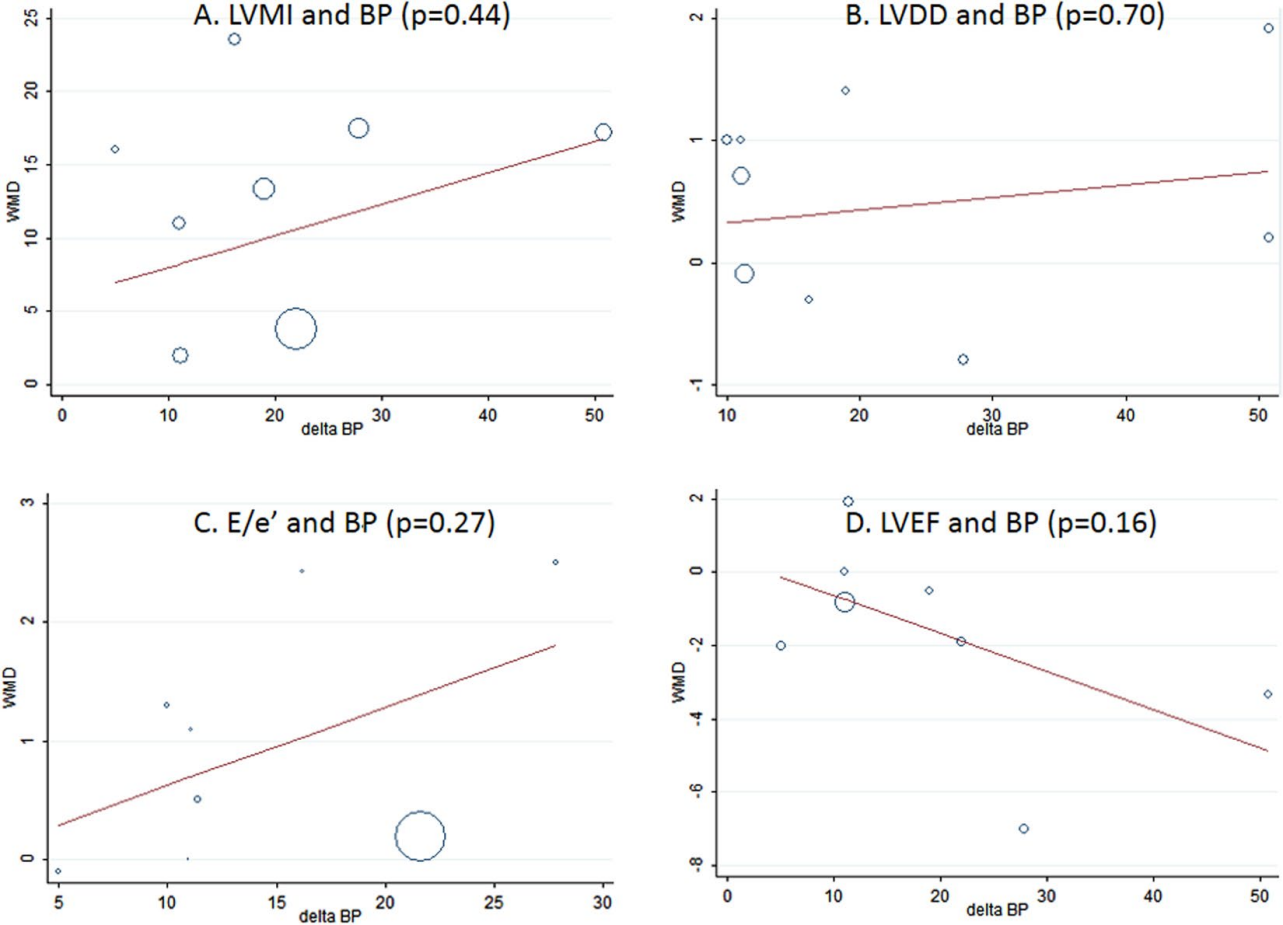
Meta-regression analysis showing the relationship between changes in (**A**) LVMI, (**B**) LVDD, (**C**) E/e’, (**D**) LVEF and systolic BP lowering.

Meta-regression analyses showed that the changes in LV remodeling or dysfunction at 6 months were not significantly associated with blood pressure lowering (Fig. 7)

## Discussion

RDN is a new interventional approach designed to treat RH. The value of RDN is still under debate, especially after failure of RDN to lower BP over and above the sham control group in the large randomized double-blinded Symplicity HTN-3 trial^28^. The secondary effects beyond BP lowering of RDN have been noticed early. The renin-angiotensin-aldosterone system (RAAS) and SNS were over-activated in patients with RH. Previous experimental studies have demonstrated that RDN could block the over-activity of SNS and RAAS, and thus exert protective effects on cardiac fibrosis and remodeling^29–31^. In 2012, Brandt *et al*. reported that RDN improved LVH and diastolic function in patients with RH using echocardiography^7^. In 2014, Mahfoud *et al*. also found that RDN reduced LVMI in a trial using CMR^18^. Notably, these effects of RDN occurred, at least in part, independently of BP^7,12^. Following these pilot studies, many trials have been conducted to investigate the effect of RDN on LVH and diastolic function.

This is the first meta-analysis on RDN and cardiac structure and function. Prior to this analysis, there have been many meta-analyses assessing the BP-lowering effects of RDN^32,33^, and only one involved the influence of RDN on LVH and atrial size^34^. That study, however, has included conference abstracts, of which the ultimate results are not available. Differing from that study, the present study included only full-text article and evaluated not only LVH but also LV diastolic function change following RDN. Furthermore, more important relevant trials, including two recent RCTs, were included in the current study, which may increase our understanding of RDN on cardiac remodeling and function.

The group of trials in the present analysis is rather heterogeneous in many respects: 1) Some trials are observational, some are RCTs; 2) Imaging was performed by CMR or echocardiography; 3) Follow-up dates ranged from 6 months to 12 months. Therefore, it is of no surprise that we separately pooled data according to study design, imaging technology and follow-up duration.

Different cardiac imaging may produce inconsistent results^35^. In our analysis, we found that both echocardiography and CMR revealed a reduction in LVMI at 6 months, indicating a consistency of echo and CMR in LVMI measurement at this time point. However, we also observed a discrepancies between echo and CMR at 12 months regarding LVMI. The small number of studies involved in CMR analysis at one year might explain this. Our study indicates a requirement for more studies with CMR analysis and long follow-up visits.

Follow-up duration is also a pivotal factor that influence the final results. We found that LVMI change at 12 months was more remarkable than that at 6 months, suggesting that the effect of RDN on LVH may be sustained up to one year. With regards to cardiac function, both E/e’ and EF were improved at 6 months, but none of them changed at 12 months. One potential explanation is that the number of trials included at 12 months was too small (only 2)^21,24^, and therefore it may be difficult to derive a conclusive analysis. More future studies should consider a longer follow up duration.

Study design is the most important factor that may impact the results. In observational studies, we found that LVH and diastolic function significantly improved, however, these results cannot be reproduced in RCTs. One reason is that observational studies have overestimated the treatment effects. Another explanation is that the two RCTs included all featured a small sample size. Large RCT remains to be needed.

There were several studies demonstrating some subgroup patients without blood pressure lowering still featured an improvement in LVMI^7,18^. Our meta-regression analysis showed that the change in LV remodeling or dysfunction were not significantly associated with blood pressure lowering (Fig. 7). These indicates that RDN might improve LV remodeling or dysfunction at least partly independent of blood pressure lowering.

The effects of RDN on HF have been frequently investigated in animal studies, most results are promising^31^. The REACH-Pilot study comprised of 7 patients showed a trend towards improvements in 6-min walk distance and diuretic use^36^. The study of Patel *et al*. included in our analysis, however, showed an unexpected negative result^26^. Nonetheless, there are several concerns regarding this trial, including small sample size, no sham-control or strict evaluation of adherence to medication and application of outdated single-electrode catheter^37^. Several ongoing studies may further increase our understanding of RDN in patients with HF^38^.

The paper of Rosa *et al*. reported one-year outcome of the randomized, controlled PRAGUE-15 study^27^, which assessed the role of adding spironolactone and RDN in RH. The authors found that RDN was not superior to intensified pharmacological treatment in improving high BP, LVH and diastolic function. Notably, the cardiac structure and function change was not the primary endpoint in the study, and sufficient number of ablations was not achieved in all patients. Similarly to the RDT-PEF trial, this study lacked a sham RDN procedure. More RCTs focusing on cardiac structure and function are required to further clarify this issue.

### Study limitations

Several drawbacks of the present study should be noticed. First, both observational studies and RCTs were included in the analysis, especially only two RCTs were finally included. Generally, RCTs constitute a higher level of data quality. Although we pooled and analyzed data from the two types of study separately, the conclusion should be interpreted with some caution. Second, despite the evaluation of diastolic function and EF, the role of RDN in HF with reduced EF was not estimated. The main reason is because there are now too few studies involving patients with reduced EF to analysis. Last, life changes may play an important role in blood pressure management, and a more complex treatment might be more effective in treating those with RH. Although RDN represents a novel and promising interventional strategy, we expect future trials to compared life changes or a more complex treatment with RDN to provide some new insights.

## Conclusions

Both LVH and cardiac function improved at 6 months following RDN. However, current evidence failed to show that RDN was superior to intensive (optimal) drug therapy in improving cardiac remodeling and function. More RCTs focusing on the impact of RDN on cardiac remodeling and function are needed. Actually, there are several ongoing trials investigating the role of RDN in cardiac remodeling and HF (NCT01870310, NCT01534299 and NCT02115230), and we expect the publication of the results of these trials to provide more information and new insights into this challenging subject.

## Electronic supplementary material

Supplementary Table 1 and 2

## References

[1] Pereira M, Lunet N, Azevedo A, Barros H (2009). Differences in prevalence, awareness, treatment and control of hypertension between developing and developed countries. J Hypertens.

[2] Gaziano TA (2005). Cardiovascular disease in the developing world and its cost-effective management. Circulation.

[3] Calhoun DA (2008). Resistant hypertension: diagnosis, evaluation, and treatment. A scientific statement from the American Heart Association Professional Education Committee of the Council for High Blood Pressure Research. Hypertension.

[4] Salles GF, Cardoso CR, Muxfeldt ES (2008). Prognostic influence of office and ambulatory blood pressures in resistant hypertension. Arch Intern Med.

[5] Kumbhani DJ (2013). Resistant hypertension: a frequent and ominous finding among hypertensive patients with atherothrombosis. Eur Heart J.

[6] Casale PN (1986). Value of echocardiographic measurement of left ventricular mass in predicting cardiovascular morbid events in hypertensive men. Ann Intern Med.

[7] Brandt MC (2012). Renal sympathetic denervation reduces left ventricular hypertrophy and improves cardiac function in patients with resistant hypertension. J Am Coll Cardiol.

[8] Inouye I (1984). Abnormal left ventricular filling: an early finding in mild to moderate systemic hypertension. Am J Cardiol.

[9] Redfield MM (2003). Burden of systolic and diastolic ventricular dysfunction in the community: appreciating the scope of the heart failure epidemic. Jama.

[10] Krum H (2009). Catheter-based renal sympathetic denervation for resistant hypertension: a multicentre safety and proof-of-principle cohort study. The Lancet.

[11] Esler MD (2010). Renal sympathetic denervation in patients with treatment-resistant hypertension (The Symplicity HTN-2 Trial): a randomised controlled trial. Lancet.

[12] Schirmer SH (2014). Improvements in left ventricular hypertrophy and diastolic function following renal denervation: effects beyond blood pressure and heart rate reduction. J Am Coll Cardiol.

[13] Liberati A (2009). The PRISMA statement for reporting systematic reviews and meta-analyses of studies that evaluate health care interventions: explanation and elaboration. PLoS Med.

[14] Berukstis A (2016). Impact of renal sympathetic denervation on cardiac sympathetic nerve activity evaluated by cardiac MIBG imaging. EuroIntervention.

[15] Dores H (2014). Renal denervation in patients with resistant hypertension: Six-month results. Revista Portuguesa de Cardiologia.

[16] Dorr O (2015). Influence of Renal Sympathetic Denervation on Cardiac Extracellular Matrix Turnover and Cardiac Fibrosis. Am J Hypertens.

[17] Ewen, S. *et al*. Blood pressure changes after catheter-based renal denervation are related to reductions in total peripheral resistance. *J*. *Hypertens*. **33**, 2519-2525 http://onlinelibrary.wiley.com/o/cochrane/clcentral/articles/911/CN-01104911/frame.html. (2015). 10.1097/HJH.0000000000000752 26485463

[18] Mahfoud F (2014). Effect of renal denervation on left ventricular mass and function in patients with resistant hypertension: data from a multi-centre cardiovascular magnetic resonance imaging trial. Eur Heart J.

[19] McLellan AJ (2015). Reverse cardiac remodeling after renal denervation: Atrial electrophysiologic and structural changes associated with blood pressure lowering. Heart Rhythm.

[20] Ripp TM (2015). Predictors of Renal Denervation Efficacy in the Treatment of Resistant Hypertension. Curr Hypertens Rep.

[21] Tsioufis, C. *et al*. Long-term effects of multielectrode renal denervation on cardiac adaptations in resistant hypertensive patients with left ventricular hypertrophy. *J Hum Hypertens*, 10.1038/jhh.2015.127 (2016). 10.1038/jhh.2015.127 26818805

[22] Tsioufis C (2015). Effects of multielectrode renal denervation on cardiac and neurohumoral adaptations in resistant hypertension with cardiac hypertrophy: an EnligHTN I substudy. J Hypertens.

[23] Verloop WL (2015). Effects of renal denervation on end organ damage in hypertensive patients. Eur J Prev Cardiol.

[24] de Sousa Almeida M (2016). Impact of Renal Sympathetic Denervation on Left Ventricular Structure and Function at 1-Year Follow-Up. PLoS One.

[25] Kiuchi MG (2016). Proof of concept study: Improvement of echocardiographic parameters after renal sympathetic denervation in CKD refractory hypertensive patients. International Journal of Cardiology.

[26] Patel, H. C. *et al*. Renal denervation in heart failure with preserved ejection fraction (RDT-PEF): a randomized controlled trial. *Eur J Heart Fail*, 10.1002/ejhf.502 (2016). 10.1002/ejhf.502 26990920

[27] Rosa J (2016). Role of Adding Spironolactone and Renal Denervation in True Resistant Hypertension: One-Year Outcomes of Randomized PRAGUE-15 Study. Hypertension.

[28] Bhatt DL (2014). A controlled trial of renal denervation for resistant hypertension. N Engl J Med.

[29] Li ZZ (2015). Renal sympathetic denervation improves cardiac dysfunction in rats with chronic pressure overload. Physiol Res.

[30] Linz D (2015). Progression of kidney injury and cardiac remodeling in obese spontaneously hypertensive rats: the role of renal sympathetic innervation. Am J Hypertens.

[31] Mahfoud F (2012). Renal hemodynamics and renal function after catheter-based renal sympathetic denervation in patients with resistant hypertension. Hypertension.

[32] Pancholy SB, Shantha GP, Patel TM, Sobotka PA, Kandzari DE (2014). Meta-analysis of the effect of renal denervation on blood pressure and pulse pressure in patients with resistant systemic hypertension. Am J Cardiol.

[33] Davis MI (2013). Effectiveness of renal denervation therapy for resistant hypertension: a systematic review and meta-analysis. J Am Coll Cardiol.

[34] Liu Q (2016). Renal denervation significantly attenuates cardiorenal fibrosis in rats with sustained pressure overload. J Am Soc Hypertens.

[35] Alfakih K, Reid S, Jones T, Sivananthan M (2004). Assessment of ventricular function and mass by cardiac magnetic resonance imaging. Eur Radiol.

[36] Davies JE (2013). First-in-man safety evaluation of renal denervation for chronic systolic heart failure: primary outcome from REACH-Pilot study. Int J Cardiol.

[37] Mahfoud F, Ewen S, Bohm M (2016). Renal denervation in patients with heart failure with preserved ejection fraction: end of the beginning?. Eur J Heart Fail.

[38] Bohm M (2014). Renal denervation and heart failure. Eur J Heart Fail.

