# Supplementary material for: Effects of Renal Denervation on Cardiac Structural and Functional Abnormalities in Patients with Resistant Hypertension or Diastolic Dysfunction
Source: Sci Rep. 2018 Jan 19;8:1172. doi: 10.1038/s41598-017-18671-6 (PMC5775308; doi:10.1038/s41598-017-18671-6)
Supplement: Supplementary file 1 — Supplementary Table 1 and 2 [file 41598_2017_18671_MOESM1_ESM.pdf]

Effects of Renal Denervation on Cardiac Structural and Functional Abnormalities in Patients with Resistant Hypertension or Diastolic Dysfunction

Short title: Renal denervation for heart remodeling and function

Total word count: 4060

Shiying Wang, MD [1,†], Suxia Yang, MD [1,†], Xinxin Zhao, MD [1], Jun Shi, MD [1,*]

[†] Shiying Wang and Suxia Yang contribute equally to the work

[1] Department of Nephrology of Huaihe Hospital of Henan University, Henan Province, China.

[*] **Corresponding author**: Jun Shi, M.D., Department of Nephrology of Huaihe hospital of Henan University, Henan Province, China

Telephone: +86- 371-3906586. **Fax:** +86- 371-3906586.

E-mail: **junshi17@yeah.net**

Author contributions statements

Shiying Wang and Suxia Yang wrote the main manuscript text; Xinxin Zhao and Jun Shi prepared figures 1–6. All authors reviewed the manuscript.

Competing Financial Interests statement: None declared.

Study or Subgroup	Post RDN Mean	Post RDN SD	Post RDN Total	Pre RDN Mean	Pre RDN SD	Pre RDN Total	Weight	Std. Mean Difference IV, Fixed, 95% CI
Brandt 2012	492	1,078	46	760	1,452	46	73.2%	-0.21 [-0.62, 0.20]
Tsioufis 2015	57.2	38.8	18	84.9	35.9	18	26.8%	-0.72 [-1.40, -0.05]
**Total (95% CI)**			**64**			**64**	**100.0%**	**-0.35 [-0.70, 0.00]**

Heterogeneity: Chi² = 1.64, df = 1 (P = 0.20); I² = 39%
Test for overall effect: Z = 1.94 (P = 0.05)

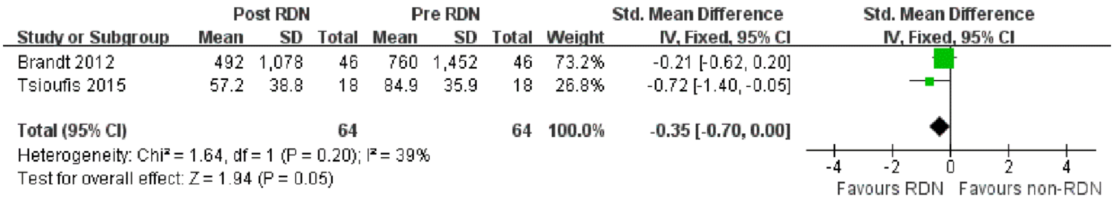

Supplementary Fig. 1. Forest plot of RDN changing BNP at 6 months in observational studies.

Supplementary Table 1. Quality assessment of included observational studies

First Author	Selection	Comparability	Outcome
Schirmer	★★★★		★★★
Ewen	★★★★		★★★
Verloop	★★★★		★★★
Berukstis	★★★★		★★★
Dorr	★★★★		★★★
Ripp	★★★★		★★★
Dores	★★★★		★★★
McLellan	★★★★		★★★
de Sousa	★★★★		★★★
Tsioufis*	★★★★		★★★
Kiuchi	★★★★		★★★
Brandt	★★★★	★★	★★★
Mahfound	★★★★	★★	★★★
Tsioufis	★★★★	★★	★★★

Quality assessment of observational studies on the basis of selection, comparability and outcome, according to the Newcastle-Ottawa Scale.

Supplementary Table 2. Quality assessment of randomized controlled trials

**Bias**	**Review authors' judgment**	**Support for judgment**
**Patel et al. study**		
Random sequence generation (selection bias)	Low Risk	Computer-generated randomisation (2:1, RD:control).
Allocation concealment (selection bias)	Low Risk	Computer-generated randomisation
Blinding of participants and personnel (performance bias)	High Risk	No clinician or patient blinding
Blinding of outcome assessment (detection bias)	Low Risk	Endpoints were analysed by observers blinded to patient allocation
Incomplete outcome data (attrition bias)	Low Risk	Only 1 patient in control group has no data on outcome of interest.
Selective reporting (reporting bias)	Low Risk	Report on all primary and secondary outcomes
Other bias	High Risk	Sample size was too small.
**Rosa et al. Study**		
Random sequence generation (selection bias)	Unclear risk	Patients…were randomized (in a 1:1 ratio) to….

		Comment: No description of the generation of the randomization
..Allocation concealment (selection bias)	Unclear risk	No description
Blinding of participants and personnel (performance bias)	High risk	No sham procedure was done in control group
Blinding of outcome assessment (detection bias)	Unclear risk	No description.
Incomplete outcome data (attrition bias)	Unclear risk	Enrollment in the study was prematurely halted (on ethical grounds)…
Selective reporting (reporting bias)	Low risk	All predefined primary and secondary outcomes were reported
Other bias	Unclear risk	Sample size was small.
